# Supplementary material for: Designing against phase and property heterogeneities in additively manufactured titanium alloys
Source: Nat Commun. 2022 Aug 9;13:4660. doi: 10.1038/s41467-022-32446-2 (PMC9363443; doi:10.1038/s41467-022-32446-2)
Supplement: Supplementary file 1 — Supplementary information [file 41467_2022_32446_MOESM1_ESM.pdf]

Supplementary information

Designing against phase and property heterogeneities in additively manufactured titanium alloys

Jingqi Zhang[1], Yingang Liu[1], Gang Sha[2*], Shenbao Jin[2], Ziyong Hou[3,4,5], Mohamad Bayat[6], Nan Yang[1], Qiyang Tan[1], Yu Yin[1], Shiyang Liu[1], Jesper Henri Hattel[6], Matthew Dargusch[1], Xiaoxu Huang[3,4*], Ming-Xing Zhang[1*]

[1]School of Mechanical and Mining Engineering, The University of Queensland, St. Lucia, Brisbane, Australia.

[2]Herbert Gleiter Institute of Nanoscience, School of Materials Science and Engineering, Nanjing University of Science and Technology, Nanjing, China.

[3]International Joint Laboratory for Light Alloys (Ministry of Education), College of Materials Science and Engineering, Chongqing University, Chongqing, China.

[4]Shenyang National Laboratory for Materials Science, Chongqing University, Chongqing, China.

[5]Department of Materials Science and Engineering, KTH Royal Institute of Technology, Stockholm, Sweden.

[6]Department of Mechanical Engineering, Technical University of Denmark, Lyngby, Denmark.

These authors contributed equally: Jingqi Zhang, Yingang Liu

[*]e-mail: gang.sha@njust.edu.cn (G.S)

xiaoxuhuang@cqu.edu.cn (X.X.H)

mingxing.zhang@uq.edu.au (M.-X.Z)

Table of Content

Designing against phase and property heterogeneities in additively manufactured titanium alloys 1

Supplementary Fig. 1 | Microstructures of Ti−6Al−4V and the newly developed 25Ti−0.25O alloy 4

Supplementary Fig. 2 | X-ray diffraction spectra of L-PBF produced Ti−6Al−4V samples taken from different locations along the building direction of the fabricated part. 5

Supplementary Fig. 3 | EBSD characterization of Ti−6Al−4V specimens tested along the vertical and horizontal directions.. 6

Supplementary Fig. 4 | Micro-CT characterization of the grip and gauge regions of Ti−6Al−4V tensile specimens after tensile testing. 7

Supplementary Fig. 5 | SEM images showing the fracture surfaces of Ti−6Al−4V tensile specimens. 8

Supplementary Fig. 6 | Calculated equilibrium phases in Ti−6Al−4V with various CP−Ti addition levels using Thermo-Calc Software.. 9

Supplementary Fig. 7 | SEM-EDS mapping of Al and V in the newly developed alloys with addition of 0.25 wt % Fe_2O_3. 10

Supplementary Fig. 8 | EBSD characterization of 25Ti−0.25O specimens tested along the vertical and horizontal directions.. 11

Supplementary Fig. 9 | Micro-CT characterization of the grip and gauge regions of tested tensile specimens of the newly developed alloys. 12

Supplementary Fig. 10 | SEM images showing the fracture surfaces of 25Ti−0.25O tensile specimens.. 13

Supplementary Fig. 11 | Microstructures of 75Ti−0.25O and 50Ti−0.25O alloys at different magnifications.. 14

Supplementary Fig. 12 | Multi-physics simulation of the L-PBF process and DICTRA simulation. 15

Supplementary Fig. 13 | Temperature dependence of diffusivities of Fe and V in β and α phases. 16

Supplementary Fig. 14 | DICTRA simulation of the composition profiles of Al and O.. 17

Supplementary Fig. 15 | TEM observation of the L-PBF fabricated 50Ti−0.5O alloy. 18

Supplementary Fig. 16 | Microstructures of 50Ti−0.25O geometrically complex component.. 19

Supplementary Fig. 17 | The meander scanning strategy used in the present work. 20

Supplementary Fig. 18 | Potentiodynamic curves for various alloys fabricated by L-PBF.....28
Supplementary Fig. 19 | Comparison of tensile properties of Ti−6Al−4V (by EB-PBF and L-PBF) and those of the newly developed alloys (by L-PBF). 31
Supplementary Fig. 20 | Microstructures of (Ti−6Al−4V + 50 wt % CP−Ti) along the building direction. 32
Supplementary Fig. 21 | Microstructures of (Ti−6Al−4V + 0.25 wt % Fe_2O_3) along the building direction.. 33
Supplementary Fig. 22 | Schematic illustration of phase transformation pathways for L-PBF produced Ti−6Al−4V and the newly developed alloys under the thermal cycling.................35
Supplementary Table 1 | Measured chemical compositions (in wt %) of as-received Ti−6Al−4V powder, CP−Ti powder and the newly designed alloy parts. 21
Supplementary Table 2 | The calculated martensite start temperature M_s for different alloys.22
Supplementary Table 3 | Thermophysical properties of the designed alloy (50Ti−0.50O) and L-PBF parameters used for the multi-physics simulation...23
Supplementary Table 4 | Corrosion parameters obtained from polarization curves. 28
Supplementary Note 1 − The reason for the highly scattered ductility in Ti−6Al−4V fabricated by L-PBF...24
Supplementary Note 2 − Addition of Fe to Ti−6Al−4V...26
Supplementary Note 3 − Addition of O to Ti−6Al−4V..29
Supplementary Note 4 − Ti−6Al−4V fabricated by EB-PBF...30
Supplementary Note 5 − The synergistic effect of CP−Ti and Fe_2O_3 additions on the phase homogeneity...31
Supplementary Note 6 – The phase transformation pathways for L-PBF produced Ti−6Al−4V and the newly developed alloys 34
Supplementary References..35

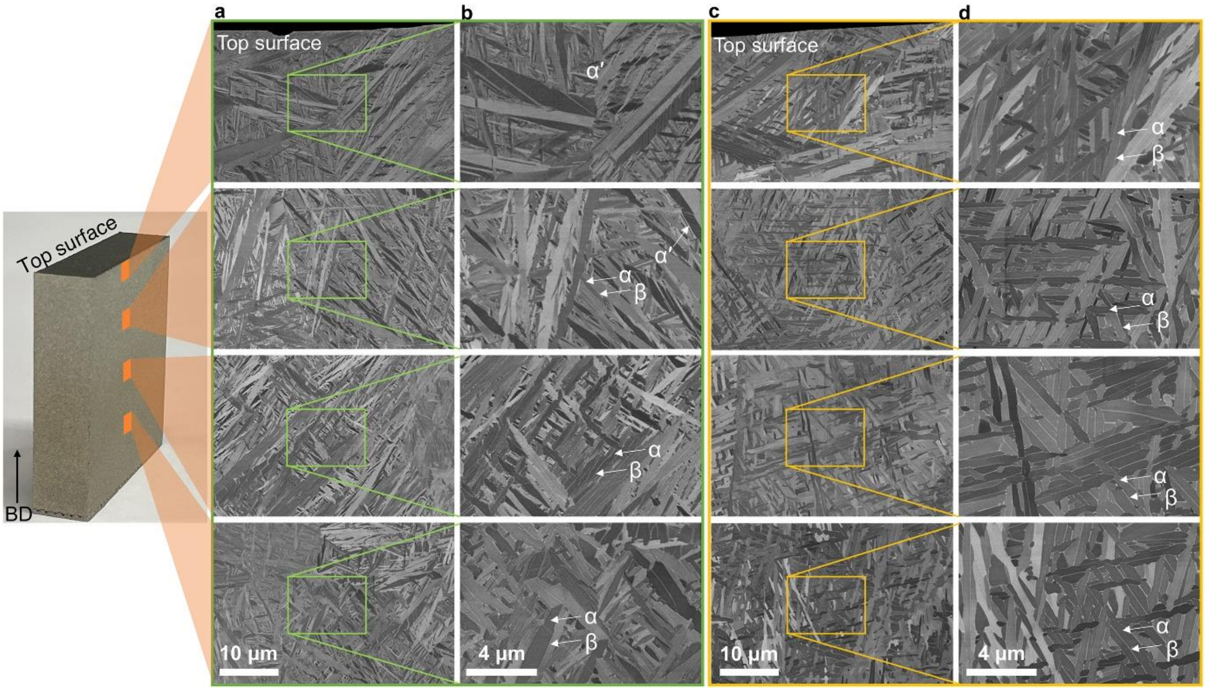

Supplementary Fig. 1 | Microstructures of Ti−6Al−4V and the newly developed 25Ti−0.25O alloy. a, SEM-BSE micrographs showing microstructures in the Ti−6Al−4V part at different locations along the building direction (BD). **b,** Higher magnification of the selected regions in **a**. The height of the L-PBF produced part is 40 mm. The distance between two characterization locations is about 7 mm. **c,** SEM-BSE micrographs showing microstructures in the 25Ti−0.25O alloy part. **d,** Higher magnification of the selected regions in **c**.

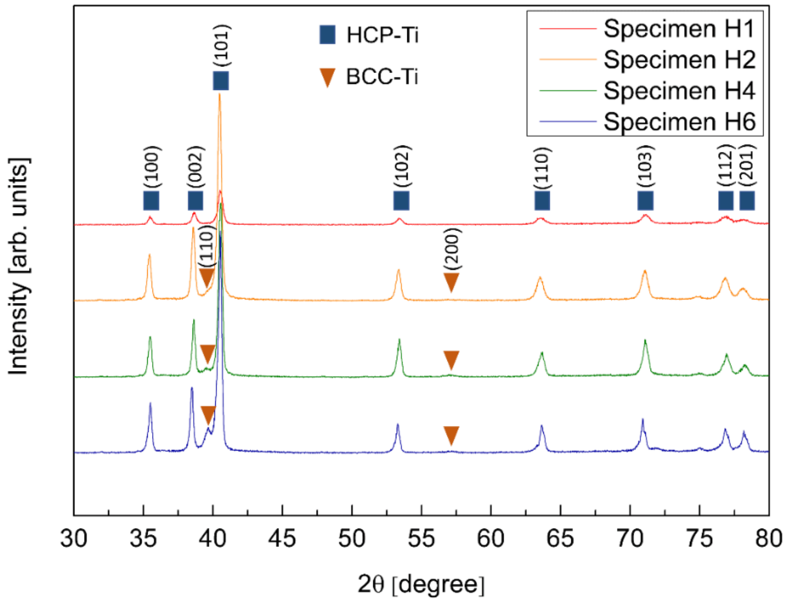

Supplementary Fig. 2 | X-ray diffraction spectra of L-PBF produced Ti–6Al–4V samples taken from different locations along the building direction of the fabricated part. It is evident that the specimen on the top surface (Specimen H1) exhibits a single hexagonal close-packed (HCP) phase while specimens in the lower regions (Specimens H2, H4 and H6) gradually show the presence of body-centred cubic (BCC) phase.

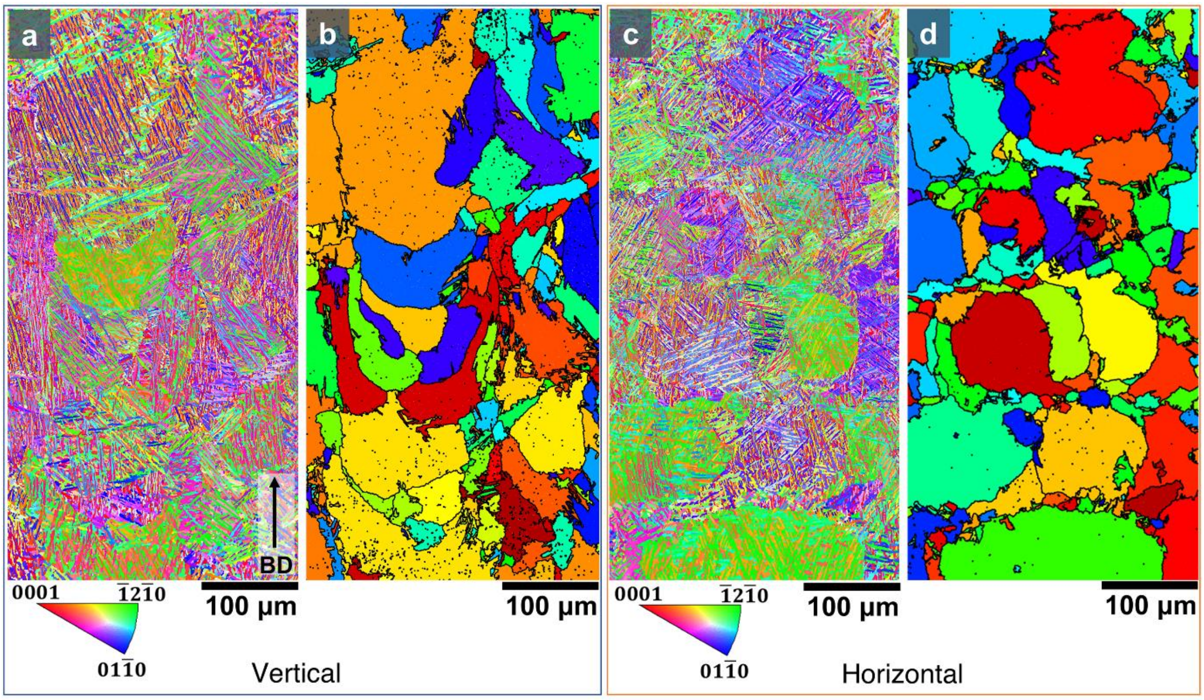

Supplementary Fig. 3 | EBSD characterization of Ti–6Al–4V specimens tested along the vertical and horizontal directions. a, EBSD inverse pole figure (IPF) of α′ and/or α phases with the hexagonal close-packed (HCP) crystal structure. The EBSD characterization was performed on Specimen V4. **b,** The reconstructed prior-β grain structure of the vertical tensile specimen. **c,** EBSD IPF of α′ and/or α phases with HCP crystal structure in the horizontal tensile specimen (Specimen H1 in Fig. 1c). **d,** The reconstructed prior-β grain structure of the horizontal tensile specimen.

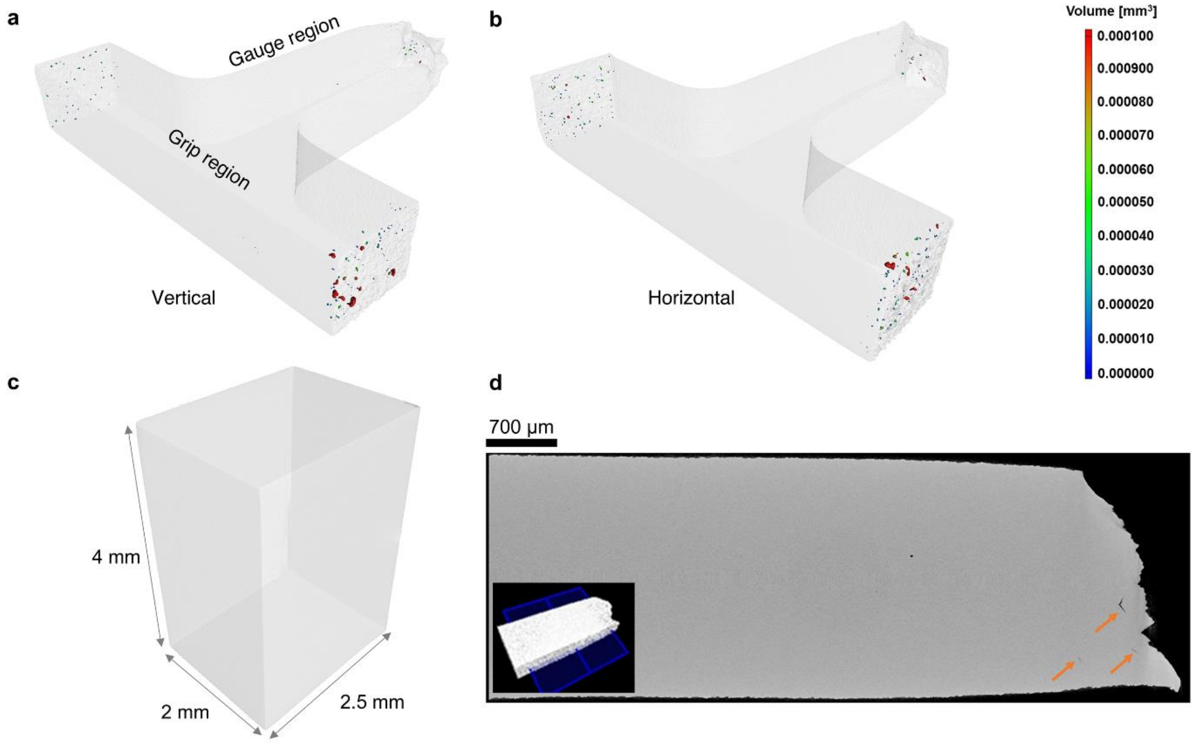

Supplementary Fig. 4 | Micro-CT characterization of the grip and gauge regions of Ti−6Al−4V tensile specimens after tensile testing. a and **b,** Micro-CT characterization of the grip and gauge regions of the Ti−6Al−4V tensile specimens tested along the vertical and horizontal directions. The vertical and horizontal specimens are V4 and H1 in Fig. 1c, respectively. **c,** Micro-CT characterization showing the grip region of Ti−6Al−4V horizontal tensile specimen at a higher scanning resolution of 2 μm. **d,** 2D Micro-CT image showing the gauge region of Ti−6Al−4V horizontal tensile specimen at a higher scanning resolution of 2 μm. The inset shows the cross section corresponding to the 2D image. The orange arrows indicate the microcracks close to the fracture surface.

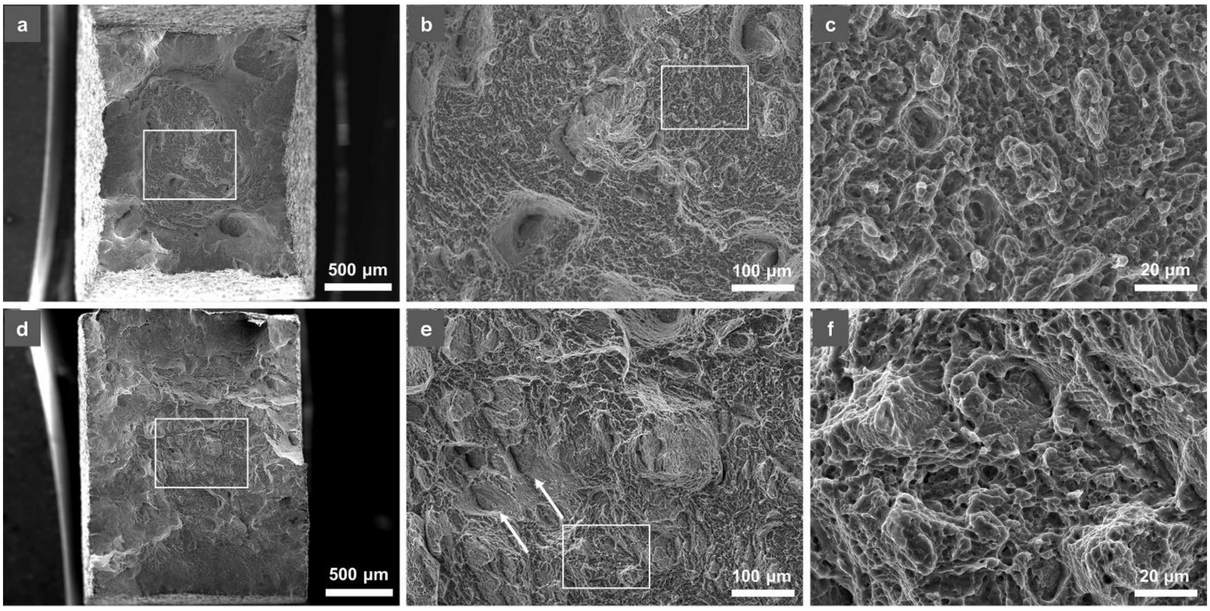

Supplementary Fig. 5 | SEM images showing the fracture surfaces of Ti−6Al−4V tensile specimens. a, The fracture surface of the vertical tensile specimen (V4 in Fig. 1c). **b** and **c,** Higher magnification images taken from the selected regions in **a** and **b**, respectively. The vertical specimen exhibits typical ductile fracture features, with a flat fibrous zone in the centre and a peripheral shear lip. **d,** The fracture surface of the horizontal specimen (H1 in Fig. 1c), showing a less ductile fracture with a limited reduction of area. **e,** Higher magnification image taken from the selected region in **d** showing considerable quasi-cleavage facets (marked with white arrows). **f,** Numerous dimples at a higher magnification of the region in the selected region in **e**.

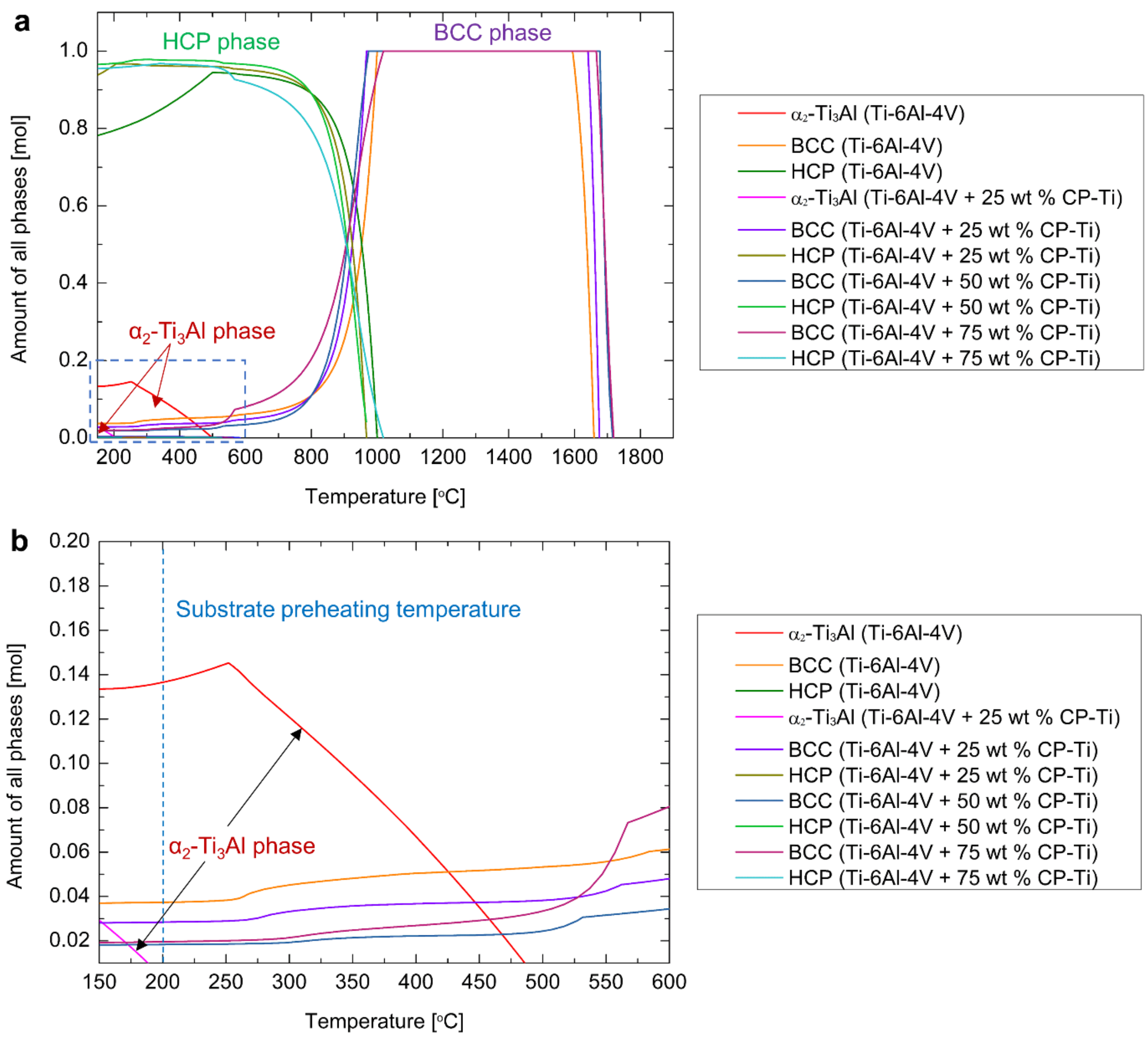

Supplementary Fig. 6 | Calculated equilibrium phases in Ti−6Al−4V with various CP−Ti addition levels using Thermo-Calc Software. a, The amount of HCP, body-centred cubic structure (BCC) and α_2-Ti_3Al phases with different additions of CP−Ti. **b,** The enlarged image of the blue dashed rectangle in **a**. It is apparent that Ti−6Al−4V shows the presence of α_2-Ti_3Al phase at around 486 °C. The addition of CP−Ti to Ti−6Al−4V significantly suppresses the formation tendency for α_2-Ti_3Al phase. The formation of α_2-Ti_3Al phase in (Ti−6Al−4V + 25 wt % CP−Ti) occurs at temperatures below 200 °C. It should be noted that the titanium substrate plate was preheated to 200 °C and the sample temperature should be above 200 °C during L-PBF due to the continuous heat input. Hence, the α_2-Ti_3Al phase in (Ti−6Al−4V + 25 wt % CP−Ti) would be suppressed during fabrication.

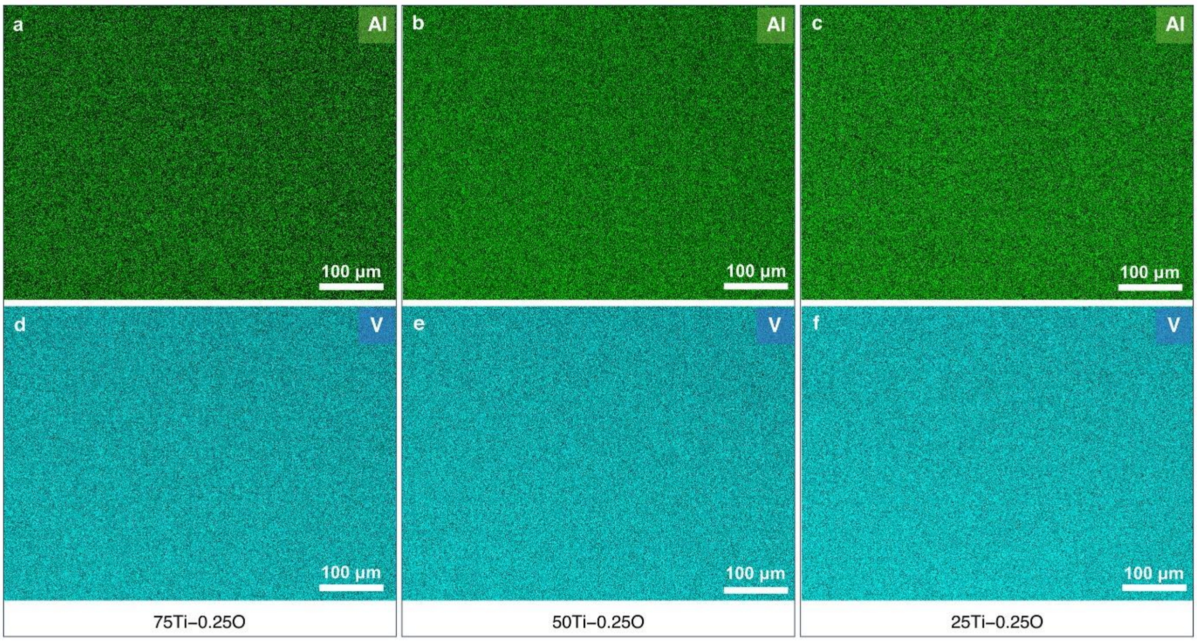

Supplementary Fig. 7 | SEM-EDS mapping of Al and V in the newly developed alloys with addition of 0.25 wt % Fe_2O_3. SEM-EDS mapping of Al in **a** 75Ti−0.25O, **b** 50Ti−0.25O, and **c** 25Ti−0.25O, respectively. SEM-EDS mapping of V in **d** 75Ti−0.25O, **e** 50Ti−0.25O, and **f** 25Ti−0.25O, respectively.

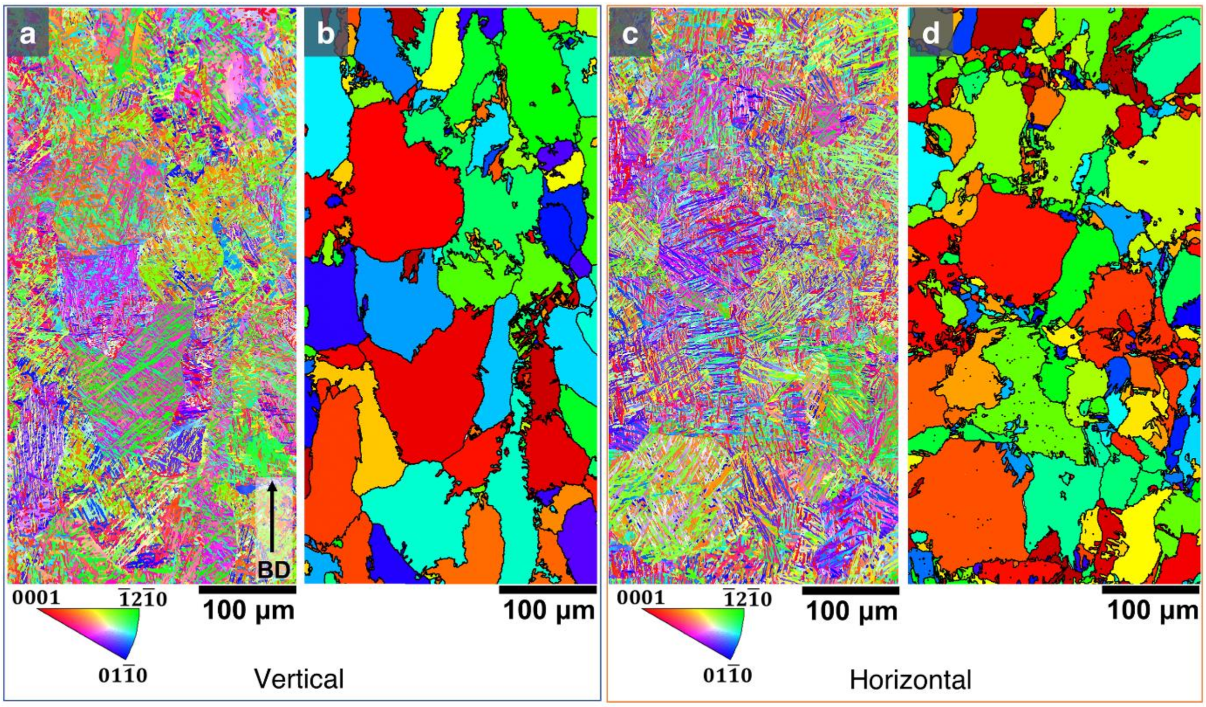

Supplementary Fig. 8 | EBSD characterization of 25Ti−0.25O specimens tested along the vertical and horizontal directions. a, EBSD IPF of α phases with HCP crystal structure. For the purpose of comparison, the EBSD characterization was also performed on Specimen V4, as shown in Fig. 1e. **b,** The reconstructed prior-β grain structure of the vertical tensile specimen. **c,** EBSD IPF of α phases with HCP crystal structure in the horizontal tensile specimen (Specimen H1 in Fig. 1e). **d,** The reconstructed prior-β grain structure of the horizontal tensile specimen.

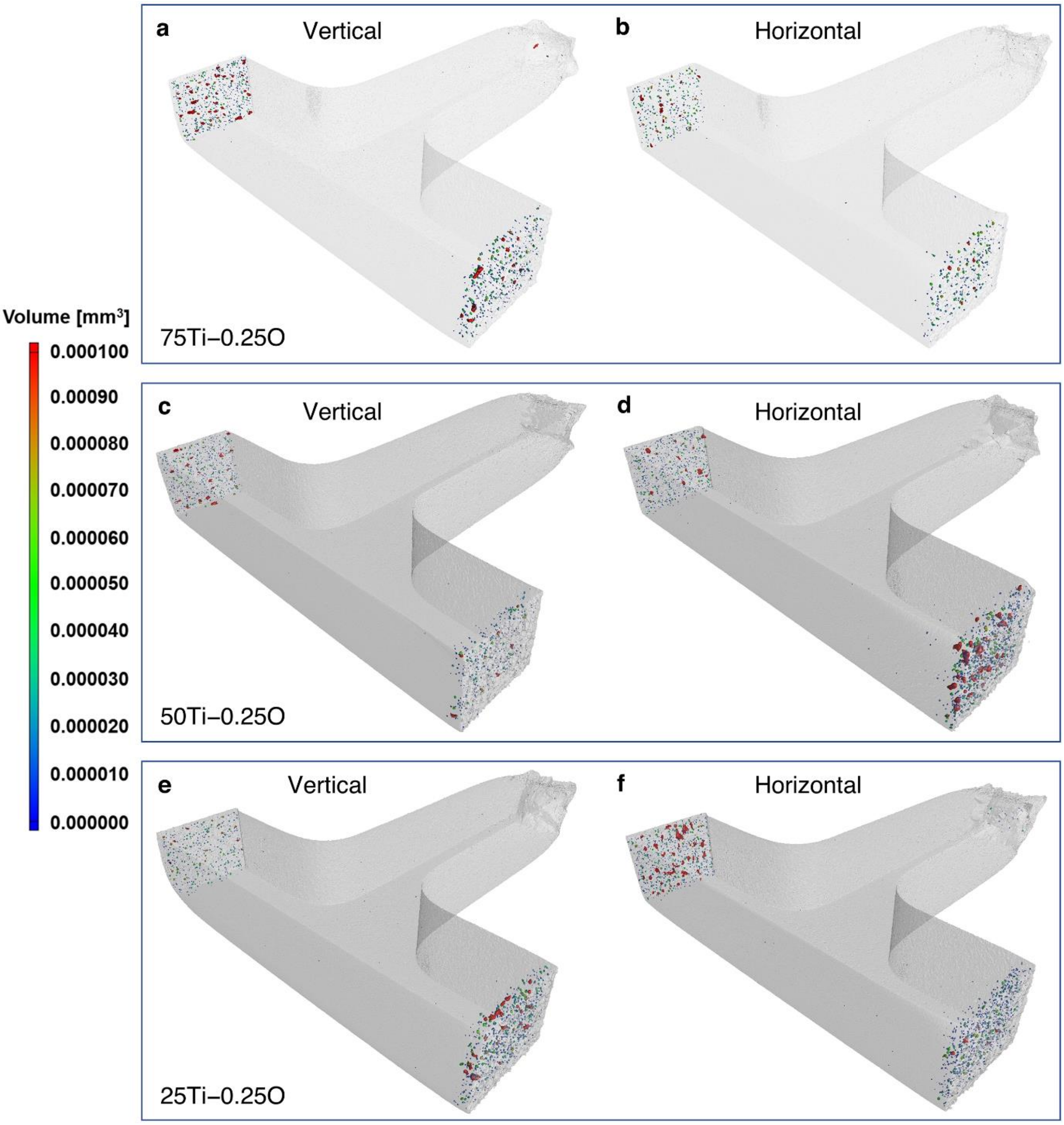

Supplementary Fig. 9 | Micro-CT characterization of the grip and gauge regions of tested tensile specimens of the newly developed alloys. a and **b,** The vertical (**a**) and horizontal (**b**) tensile specimens of 75Ti−0.25O. **c** and **d,** The vertical (**c**) and horizontal (**d**) tensile specimens of 50Ti−0.25O. **e** and **f,** The vertical (**e**) and horizontal (**f**) tensile specimens of 25Ti−0.25O. Note that the vertical and horizontal specimens are V4 and H1 in Fig. 1e, respectively. It shows that there are a few pores at the edges of the grip region, due to a default processing parameter used in the part borders. This is similar to that observed in the Ti−6Al−4V specimens.

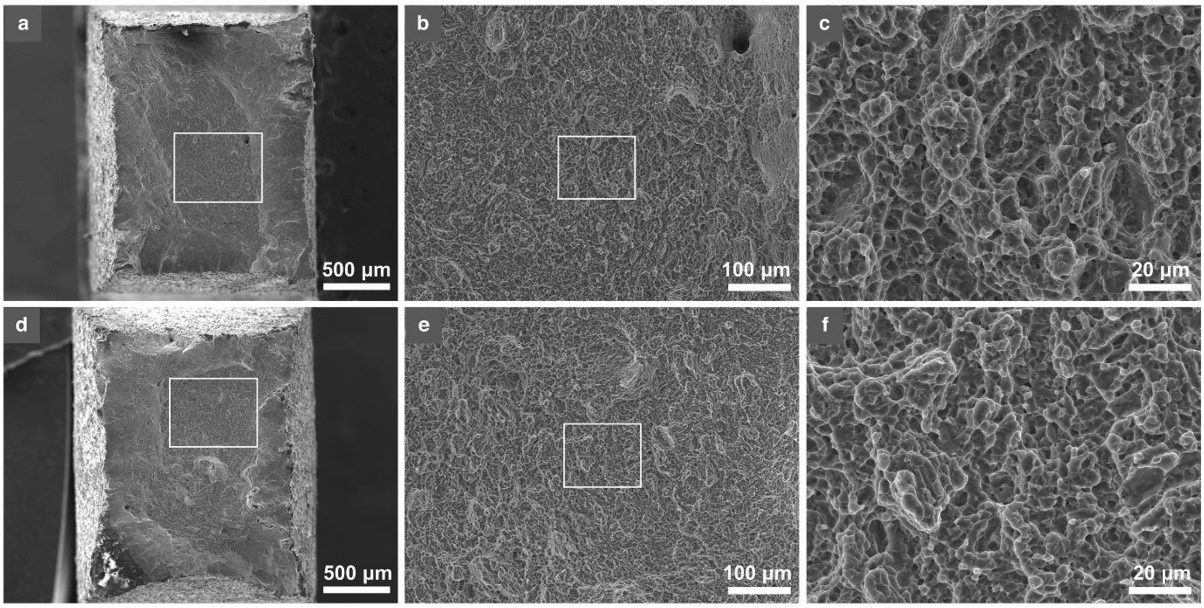

Supplementary Fig. 10 | SEM images showing the fracture surfaces of 25Ti−0.25O tensile specimens. a, The fracture surface of the vertical tensile specimen (V4 in Fig. 1e). **b** and **c**, Higher magnification images taken from the selected regions in **a** and **b**, respectively. **d,** The fracture surface of the horizontal tensile specimen (H1 in Fig. 1e). **e** and **f**, Higher magnification images taken from the selected regions in **d** and **e**, respectively. Unlike Ti−6Al−4V, the newly developed 25Ti−0.25O alloy exhibits essentially similar fracture features in both the vertical and horizontal tensile specimens. It can be seen that both specimens show a significant reduction of area and numerous dimples at a higher magnification, indicating a typical ductile fracture. Additionally, the horizontal specimen does not show any quasi-cleavage facets, as observed in the case of Ti−6Al−4V.

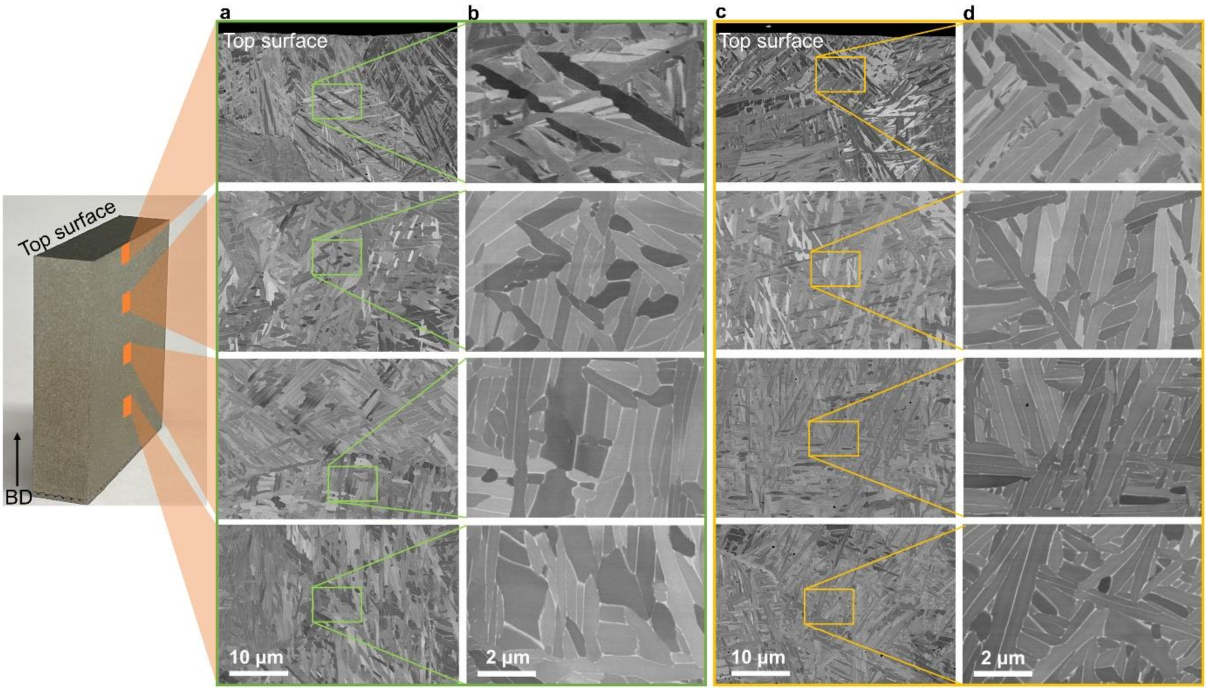

Supplementary Fig. 11 | Microstructures of 75Ti−0.25O and 50Ti−0.25O alloys at different magnifications. a, SEM-BSE micrographs of different locations in the 75Ti−0.25O part along the building direction (BD). **b,** Higher magnification of the selected regions in **a**. The height of the L-PBF produced part is 40 mm. The distance between two characterization locations is about 7 mm. **c,** SEM-BSE images of different locations in the 50Ti−0.25O alloy part. **d,** Higher magnification of the selected regions in **c**.

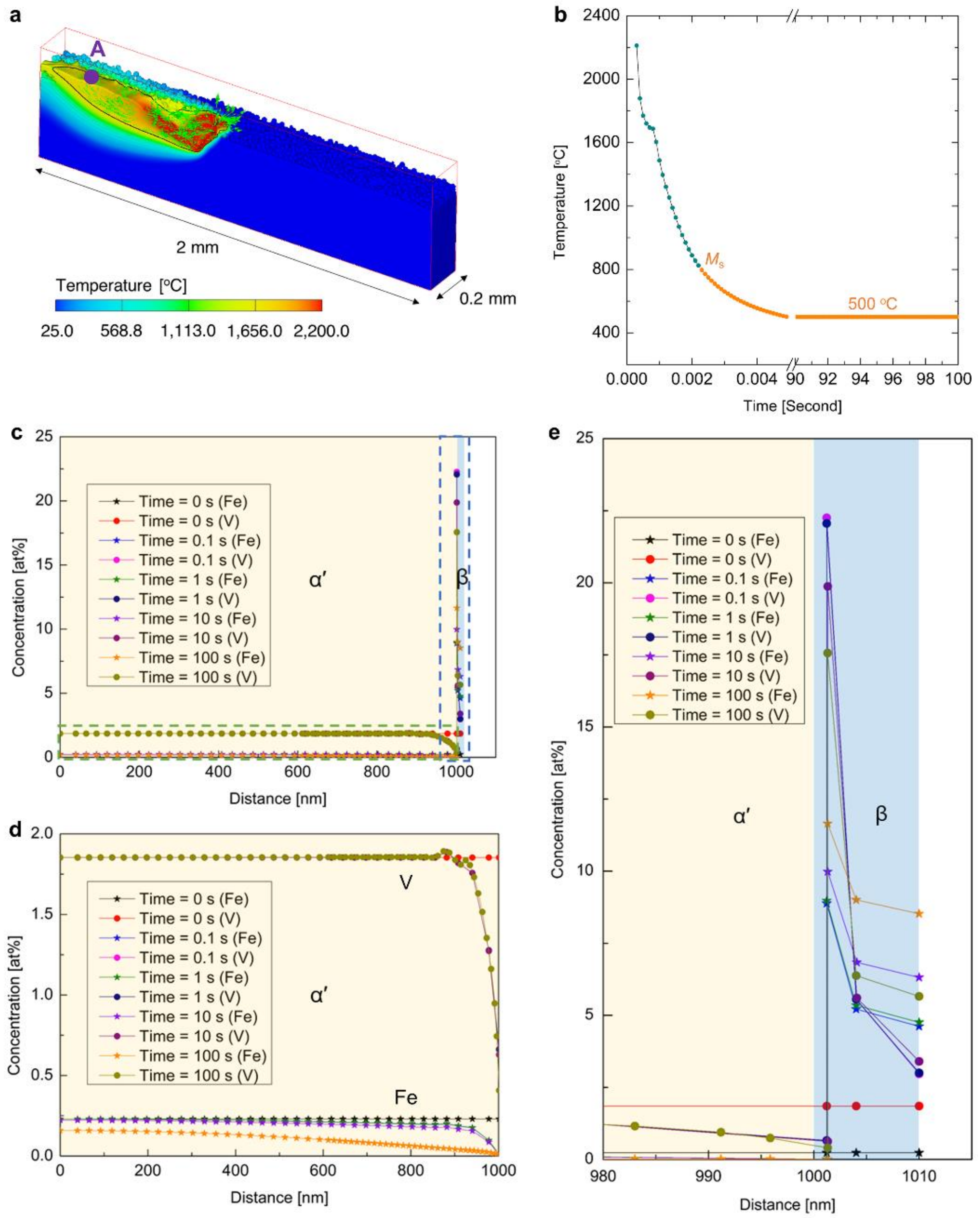

Supplementary Fig. 12 | Multi-physics simulation of the L-PBF process and DICTRA simulation. a, 3D view of the multi-physics simulation domain of the L-PBF process. **b,** The cooling curve of point A on the top surface in **a**. The cooling profile that starts from M_s to 500 °C and then maintains at 500 °C until 100 s is used for DICTRA simulation, as highlighted in the cooling curve with orange data points. **c,** DICTRA simulation of composition profiles of Fe and V across the α′/β interface at various times under the cooling process (without laser re-melting). **d,** Magnified view of the α′ phase simulation domain (marked with green dashed rectangle in **c**), showing that Fe and V diffuse out from the α′ and the diffusion kinetics of Fe is faster than those of V. **e,** Magnified view of β phase simulation domain marked with blue dashed rectangle in **c**, showing the accumulation of Fe and V in the β phase.

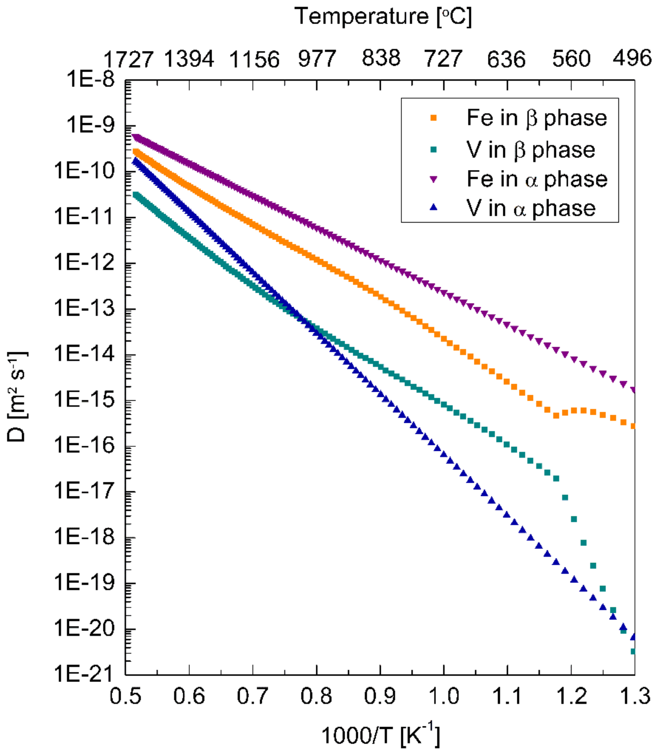

Supplementary Fig. 13 | Temperature dependence of diffusivities of Fe and V in β and α phases. The diffusivity values of Fe and V were calculated using Thermo-Calc software implemented TCTI3 and MOBTI4 database.

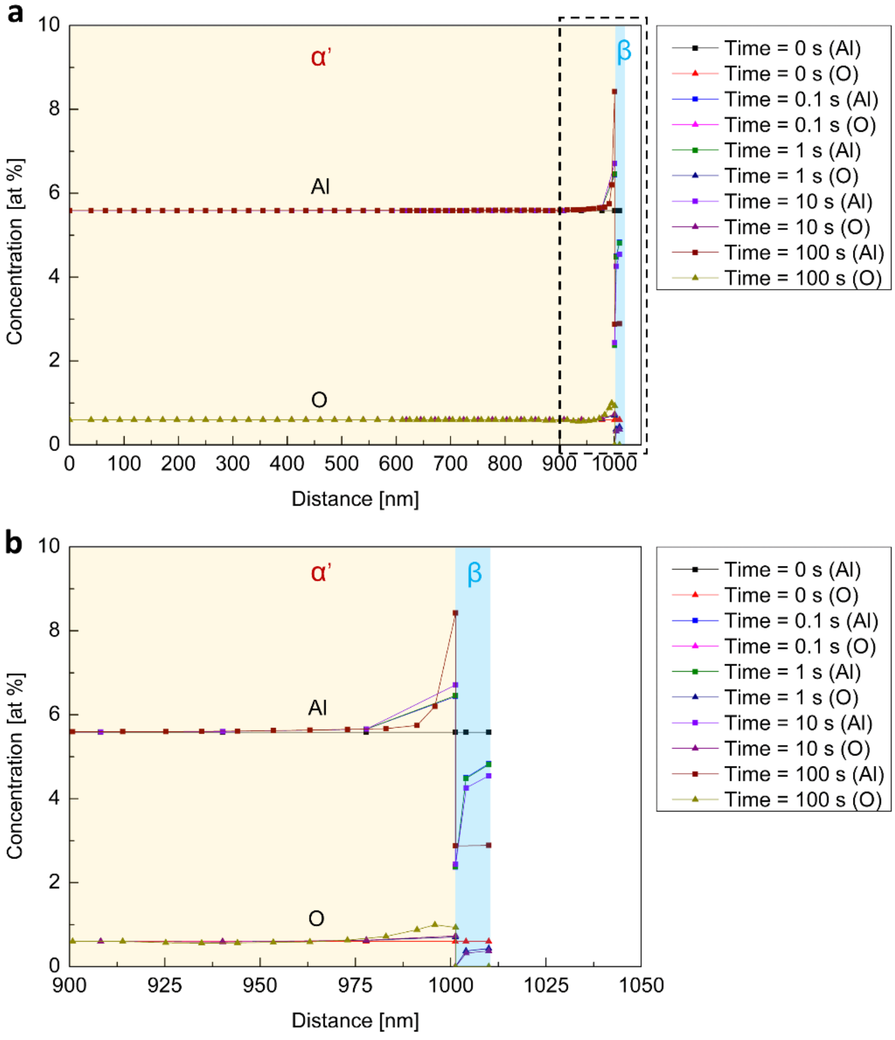

Supplementary Fig. 14 | DICTRA simulation of the composition profiles of Al and O. a, composition profiles of Al and O across the α′/β interface at various times. **b**, Magnified view of the α′ and β phase simulation domains (marked with black dashed rectangle in **a**) showing the accumulation of Al and O in the α′ phase.

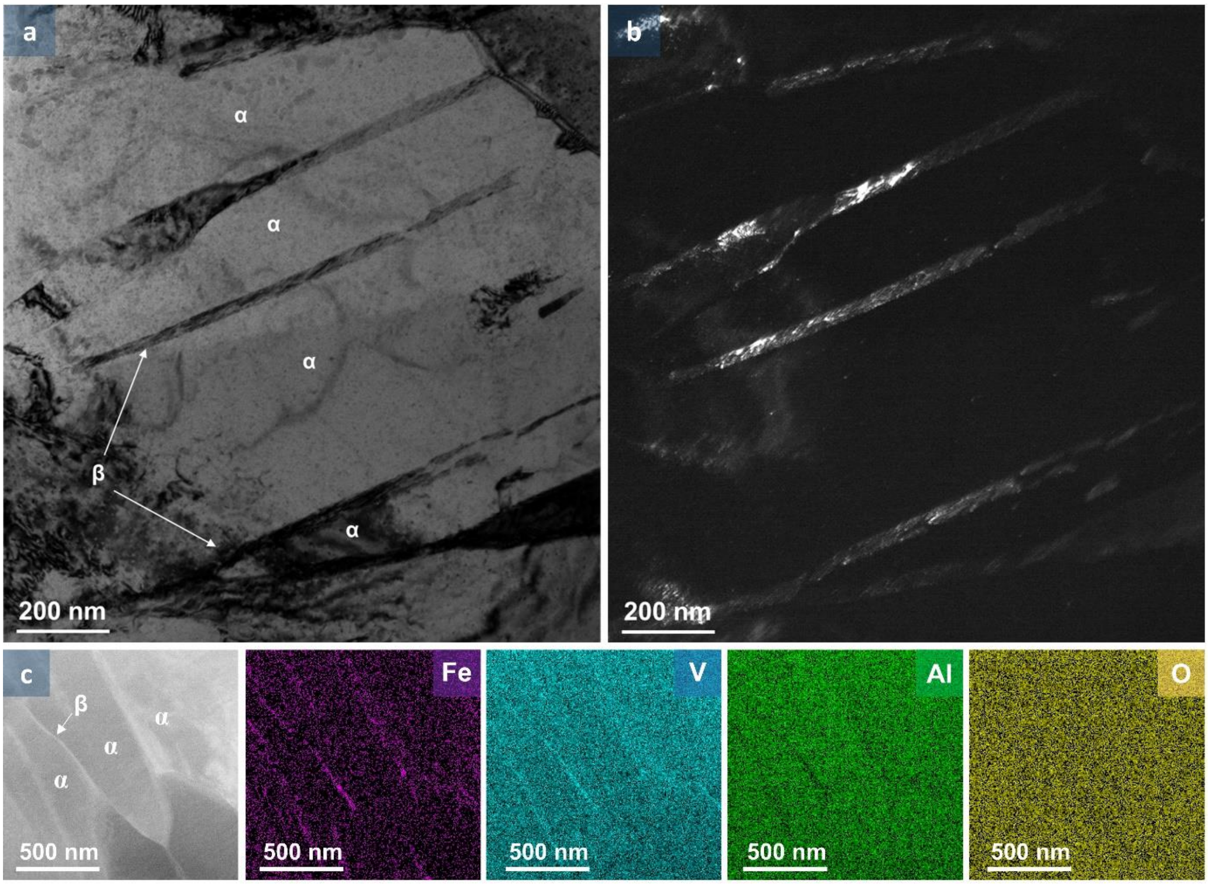

Supplementary Fig. 15 | TEM observation of the L-PBF fabricated 50Ti−0.50O alloy. **a**, Bright-field TEM image showing the presence of fine β plates between coarse α laths. **b**, Dark-field image corresponding to **a** showing the β phase. **c**, HADDF-STEM image and EDS elementary mapping (Fe, V, Al and O).

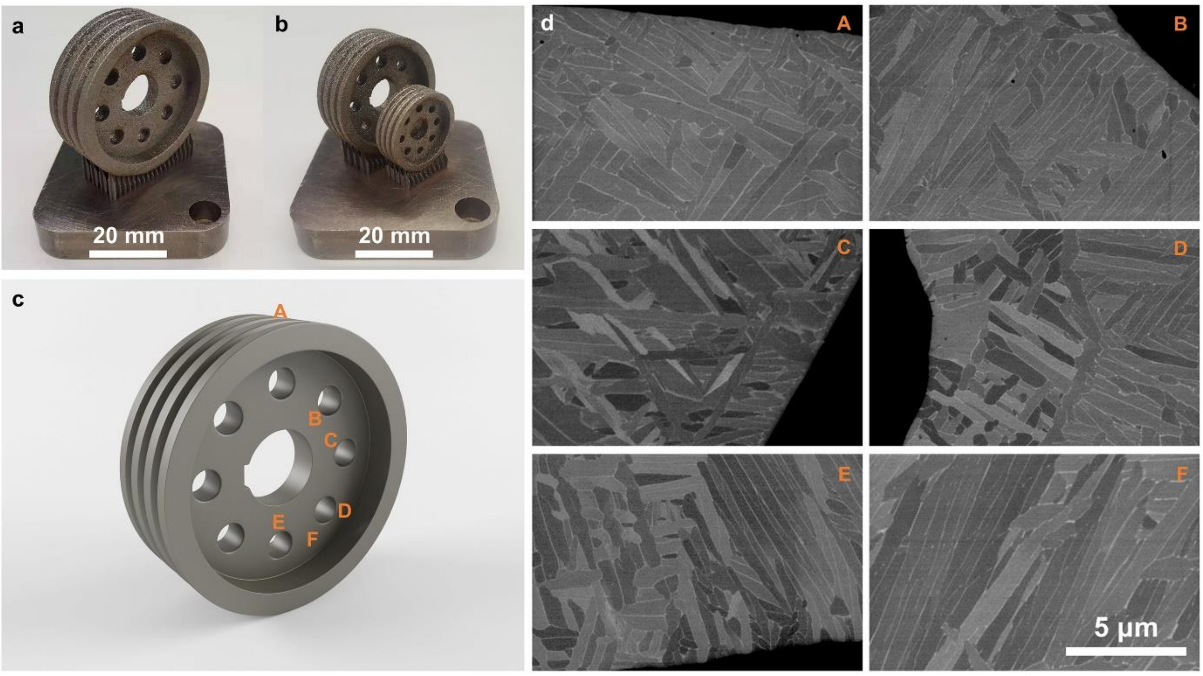

Supplementary Fig. 16 | Microstructures of 50Ti−0.25O geometrically complex component. a and **b,** Components with complex geometry produced by L-PBF. **c,** The model showing the locations of microstructural examination of the component with smaller size in **b**. **d,** Lamellar (α+β) microstructures in different locations marked in **c**.

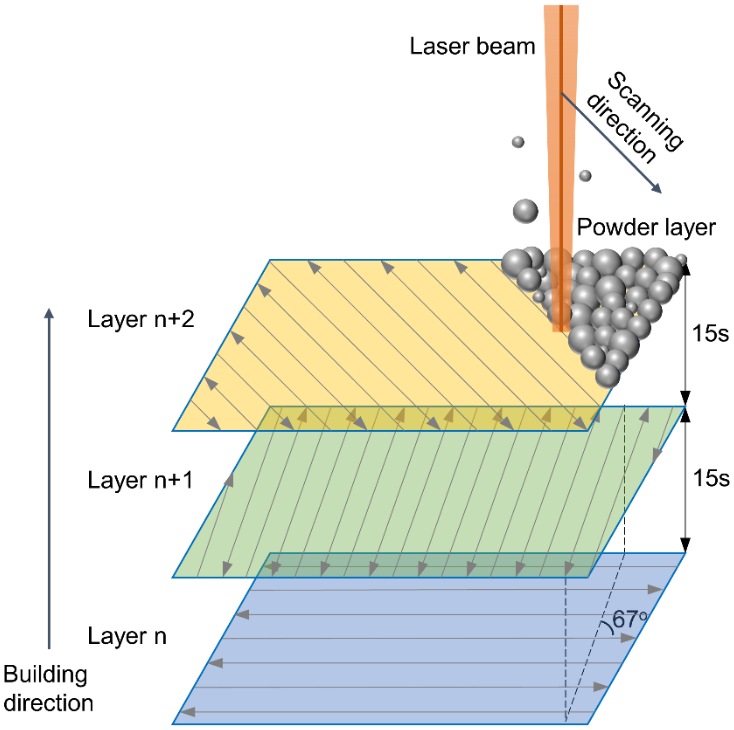

Supplementary Fig. 17 | The meander scanning strategy used in the present work. Note that the rotation angle and the time interval between two successive layers are 67° and 15 s, respectively.

Supplementary Table 1 | Measured chemical compositions (in wt %) of as-received Ti−6Al−4V powder, CP−Ti powder and the newly designed alloy parts.

Material	Al	V	Fe	O	N	C	H	Ti
Ti−6Al−4V	6.48	4.06	0.21	0.08	0.01	0.02	0.003	Bal.
CP−Ti	/	/	0.04	0.18	0.01	0.02	0.002	Bal.
75Ti−0.25O	1.54	1.07	0.30	0.32	0.056	0.02	0.005	Bal.
50Ti−0.25O	3.08	2.05	0.33	0.30	0.040	0.01	0.006	Bal.
50Ti−0.50O	3.08	2.06	0.48	0.42	0.063	0.02	0.006	Bal.
25Ti−0.25O	4.62	3.03	0.38	0.28	0.060	0.02	0.006	Bal.
25Ti−0.50O	4.80	3.20	0.50	0.40	0.062	0.02	0.008	Bal.

Supplementary Table 2 | The calculated martensite start temperature M_s for different alloys.

Alloys based on different powder mixtures		M_s [ºC]
Ti−6Al−4V [wt %]	CP−Ti [wt %]	
75	25	784.6
50	50	819.8
25	75	844.1

Supplementary Table 3 | Thermophysical properties of the designed alloy (50Ti−0.50O) and L-PBF parameters used for the multi-physics simulation.

Parameter	Symbol	Value	Unit
Laser power	P	350	W
Scanning speed	v	1,400	mm s^{-1}
Effective radius of beam	r_b	40	μm
Substrate temperature	Ts	298	K
Boiling temperature	T_{boil}	3,480	K
Liquidus temperature	T_l	1,965	K
Solidus temperature	T_s	1,936	K
Specific heat capacity of liquid	$C_{p,l}$	697	J $kg^{-1}\cdot K^{-1}$
Specific heat capacity of solid	$C_{p,s}$	501	J $kg^{-1}\cdot K^{-1}$
Thermal conductivity of liquid	k_l	28	W $m^{-1}\cdot K^{-1}$
Thermal conductivity of solid	k_s	17.5	W $m^{-1}\cdot K^{-1}$
Density of liquid	ρ_l	4,000	kg m^{-3}
Density of solid	ρ_s	4,347	kg m^{-3}
Liquid surface tension	σ_0	1.407	N m^{-1}
Surface tension sensitivity to temperature	γ	-0.000149	N $m^{-1}\cdot K^{-1}$
Latent heat of fusion	ΔH_{sl}	361	kJ kg^{-1}
Latent heat of evaporation	ΔH_{lv}	8.17	MJ kg^{-1}

Supplementary Note 1 − The reason for the highly scattered ductility in Ti−6Al−4V fabricated by L-PBF

To uncover the origin of the dramatic scattering in ductility of L-PBF produced Ti−6Al−4V, we carried out detailed microstructural analyses using EBSD, SEM and Micro-CT. It is well recognized that columnar grains and porosity are the most common fingerprints for mechanical inhomogeneity[1,2]. In this study, we have refined the L-PBF processing parameters using Ti−6Al−4V powders, with the aim of minimizing porosity and, more importantly, breaking up the columnar prior-β grains. We first focus on the grain structure of Ti−6Al−4V. The EBSD inverse pole figures (IPF) of the vertical and horizontal specimens are shown in Supplementary Fig. 3a and c, respectively. It can be seen that both specimens are characterized by needle-like hexagonal closed-packed (HCP) phases. The prior-β grains were reconstructed from the EBSD data using the ARPGE software, as presented in Supplementary Fig. 3b,d, respectively. It can be observed that the prior-β grains in both specimens are in the irregular shape but do not exhibit the columnar grain morphology. Hence, the grain structure of Ti−6Al−4V is unlikely responsible for the pronounced mechanical inhomogeneity observed here.

We then turn our attention to the porosity. Pores are commonly introduced during the L-PBF process. Additionally, Voisin et al.[3] have demonstrated that another type of pores (that is, "edge-of-track" pores) can form during L-PBF. Such small pores are typically formed along the edges of melt track. Upon subjected to tension, they can grow and coalesce, thereby resulting in the scattering of mechanical properties. In this work, Micro-CT characterizations were carried out on the grip and gauge regions of the tested tensile specimens, which allowed for direct observation of the porosity distribution in both as-built and post-tension states (Supplementary Fig. 4a). In the grip regions of both specimens, a number of visible pores are

mainly distributed in the edge area (Supplementary Fig. 4a,b). This is attributed to the manufacturer's default processing parameters (that is, laser power of 100 W and scanning speed of 450 mm s^{-1}) that were used to create the borders of Ti−6Al−4V parts. It should be noted that such pores do not affect the final tensile results, because the borders in the gauge region have been machined away according to the geometry of tensile specimen. Under the refined processing conditions, only a few micropores are detected in other areas, indicating a very high density of Ti−6Al−4V parts in the as-built state. While in the gauge region, a number of pores are found near the fracture surface, particularly in the central region (Supplementary Fig. 4b). Such pores seem unlikely to be produced during fabrication, because they do not distribute in other areas that are far from the fracture surface, but rather were induced in the deformation process. It is well documented that the flat fibrous zone of the fracture surface is typically associated with pore nucleation, growth and coalescence[4].

Owing to the large size of specimens, the resolution of Micro-CT characterization is limited to be 4 μm. In order to achieve a higher resolution, the selected areas in the grip and gauge regions of the horizontal specimen H1 were characterized, respectively. This allows for a higher Micro-CT resolution of 2 μm. In the selected grip region (Supplementary Fig. 4c), no porosity is detected under the present resolution limit. In the case of the gauge region (Supplementary Fig. 4d), a few microcracks (as marked with arrows) oriented approximately 45° with respect to the loading direction are found close to the fracture surface. This suggests that the nucleation and coalescence of such microcracks may lead to the final rupture. Similar 45°-oriented microcracks have also been reported by Moridi et al.[5], who have shown that strain localization mainly occurs in the long primary α′ martensite that forms an angle of about 45° with loading direction, thereby serving as a precursor to crack initiation. This is in line with the observation of the fracture surfaces, as will be discussed below.

The fracture surfaces of tested specimens were then observed by SEM to gain a deeper insight into the fracture mechanism, as shown in Supplementary Fig. 5. The vertical specimen exhibits typical ductile features, with a flat fibrous zone in the center and a peripheral shear lip (Supplementary Fig. 5a-c). By contrast, the horizontal specimen shows a less ductile fracture surface with a limited reduction of area (Supplementary Fig. 5d). Additionally, higher magnification of the fracture surface shows considerable quasi-cleavage facets (Supplementary Fig. 5e). Similar fracture features have been reported in other study on additively manufactured Ti−6Al−4V by L-PBF, and are attributed to the crack propagation along the long martensite colony[6]. The fracture surface analysis is consistent with the Micro-CT characterization (Supplementary Fig. 4), indicating that martensite could be the dominant factor behind the less ductile mechanical response of horizontal specimen H1. This is further supported by the phase analysis along the building direction in the main text (Fig. 1b and Supplementary Fig. 1b).

In summary, the microstructural analysis suggests that the phase inhomogeneity along the building direction, rather than the grain structure or porosity, is the most plausible cause of spatially dependent ductility in Ti−6Al−4V produced by L-PBF in this work.

Supplementary Note 2 − Addition of Fe to Ti−6Al−4V

There have been a few studies on the addition of Fe to Ti−6Al−4V, aiming at eliminating the mechanical anisotropy through refining the prior-β grain morphology[7,8]. However, the design strategies in these studies are quite different from the design approach demonstrated in the present work.

The key to these studies lies in the relatively high growth restriction effect of Fe in Ti during the solidification of AM. The degree of growth restriction for a given solute can be described by the growth restriction factor (Q), which is defined as[9,10,11]:

$$Q = C_s m(k-1) \qquad (1)$$

where C_s is the solute content, m is the slope of the equilibrium liquidus and k is the equilibrium partition coefficient. Based on the growth restriction theory, Simonelli et al.[7] showed that the addition of 3 wt % Fe to Ti−6Al−4V by L-PBF led to a mixed columnar and apparent equiaxed prior-β grains morphology but failed to produce truly randomly oriented equiaxed grains. Therefore, the risk of mechanical inhomogeneity may remain, although no mechanical property was provided in their study. Narayana et al.[8] found that addition of 3 wt % or higher Fe to Ti−6Al−4V by DED was detrimental to the tensile ductility, resulting in a reduced elongation to failure below 5%. This is consistent with the study by Alabort et al.[12], who suggested a critical threshold (Fe + Ni + Cr $\leqslant$ 3.0 wt %) as a guideline to avoid the brittle intermetallic in titanium alloys.

In this work, we attempted to tailor the constituent phases within the prior-β grains through solid-state phase transformations, rather than to promote the columnar-to-equiaxed transition of prior-β grains due to the growth restriction effect of Fe in solidification. The trace addition levels of Fe used in this work (Supplementary Table 1) are far below the recommended critical threshold of Fe in titanium alloys (that is, 3 wt %[12]). Such low Fe additions do not have an apparent impact on the morphology of prior-β grains (Supplementary Fig. 3 and Supplementary Fig. 8). In fact, in the present work, the columnar prior-β grains, as frequently seen in additively manufactured Ti−6Al−4V, have been eliminated through optimizing the processing parameters. This distinguishes our work from existing studies that consider the columnar prior-β grains as the dominant factor responsible for the microstructural heterogeneity in titanium alloys. Since the mechanical properties of a wide variety of metallic materials depend largely on not only the grain size but also the phase constitution, eliminating the phase heterogeneity − which is of

equal, if not greater, importance to controlling the grain structure − remains a great challenge in achieving uniform mechanical properties, which is the focus of the present work.

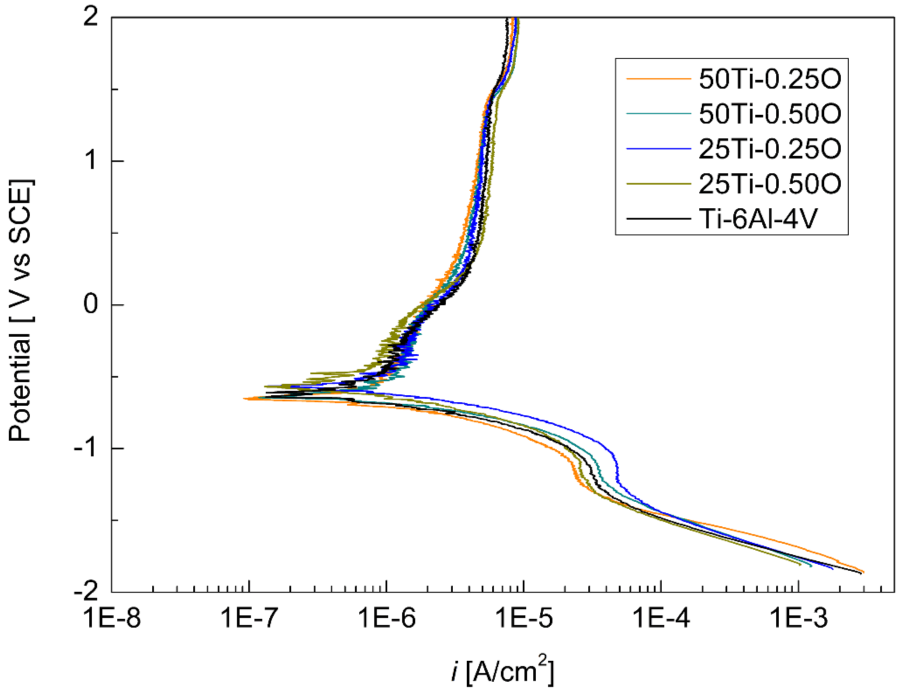

Supplementary Fig. 18 | Potentiodynamic curves for various alloys fabricated by L-PBF. The electrochemial measurements were carried out in 3.5 wt % NaCl solution.

Supplementary Table 4 | Corrosion parameters obtained from polarization curves.

Alloy	E_{corr} [V]	i_{corr} [μA cm^{-2}]	E_p [V]	i_p [μA cm^{-2}]
50Ti−0.25O	– 0.66 ± 0.11	0.75 ± 0.04	0.30 ± 0.03	4.34 ± 0.04
50Ti−0.50O	– 0.61 ± 0.08	0.74 ± 0.06	0.31 ± 0.05	4.49 ± 0.09
25Ti−0.25O	– 0.59 ± 0.09	0.72 ± 0.07	0.33 ± 0.06	4.86 ± 0.06
25Ti−0.50O	– 0.66 ± 0.07	0.67 ± 0.11	0.37 ± 0.08	5.42 ± 0.10
Ti−6Al−4V	– 0.65 ± 0.09	0.77 ± 0.09	0.32 ± 0.05	5.10 ± 0.07

In addition to its negative effect on the ductility, high Fe content can degrade the corrosion resistance of Ti−6Al−4V[13]. In this work, we evaluated the corrosion resistance of the newly developed alloys in 3.5 wt % NaCl solution and compared it with that of Ti−6Al−4V. Supplementary Fig. 18 shows the potentiodynamic polarization curves for Ti−6Al−4V and our

selected alloys (50Ti−0.25O, 25Ti−0.50O, 25Ti−0.25O and 25Ti−0.50O) that have relatively high strength. It is shown that all alloys exhibit an essentially similar corrosion behavior and an evident passivation region at the potential above 0.5 V, where the current density changes slightly with increasing potential. The corrosion parameters − that is, corrosion potential (E_{corr}), corrosion current density (i_{corr}), passivation potential (E_p) and passivation current density (i_p) − of each alloy are determined from the potentiodynamic polarization curves, as listed in Supplementary Table 4. Overall, all alloys show very close values of E_{corr}, i_{corr} and E_p but a relatively obvious change in i_p. Additionally, the newly developed alloys except for 25Ti−0.25O exhibit a lower i_p than Ti−6Al−4V. i_p represents the passivation current for the protective film formed on the sample surface and a lower i_p indicates better corrosion performance[14]. Besides, for a given CP−Ti addition, increasing Fe_2O_3 addition level increase i_p. This trend is in line with the study by Zhao et al.[13], which shows that increasing Fe addition leads to a higher i_p. This also confirms that high Fe addition to titanium degrades the corrosion resistance. Nevertheless, the 25Ti−0.25O alloy exhibits a very limited reduction in the corrosion resistance compared with Ti−6Al−4V, due to the trace Fe addition level.

Supplementary Note 3 − Addition of O to Ti−6Al−4V

Oxygen (O) is commonly known as an effective strengthening agent for titanium but may have a detrimental impact on the tensile ductility of some titanium alloys such as Ti−6Al−4V. In this work, our alloy design strategy allows for a higher O addition in the newly developed alloys than Ti−6Al−4V while keeping good tensile ductility in two ways. On the one hand, the addition of CP−Ti to Ti−6Al−4V enhanced the oxygen tolerance in the newly developed alloys. It has been shown that CP−Ti has a notably higher critical O content than Ti−6Al−4V to maintain good tensile ductility (for example, 10%)[15]. On the other hand, the combined additions of CP−Ti and Fe_2O_3 to Ti−6Al−4V resulted in the lamellar (α+β) phases. The

detrimental effect of O on the tensile ductility is microstructural dependent. It has been found that additively manufactured Ti−6Al−4V with the lamellar (α+β) microstructure has a significantly higher critical O content (that is, up to 0.36 wt %) than that consisting of α′ martensite (0.22−0.25 wt %)[15]. Overall, from the compositional and microstructural perspectives, this rationalizes that our alloys can tolerate O level higher than 0.40 wt % while still achieving good tensile ductility above 10%.

Supplementary Note 4 − Ti−6Al−4V fabricated by EB-PBF

EB-PBF, commonly known as electron beam melting (EBM), enables in-situ martensite decomposition in Ti−6Al−4V by heating the substrate plate to temperatures above the α′ martensitic transformation temperature[16,17]. However, it has been shown that the microstructural heterogeneity cannot be entirely eliminated through EB-PBF. Tan et al.[17] found that the grain structure of prior-β grains of the EB-PBF produced Ti−6Al−4V changes from near-equiaxed to columnar morphology along the building direction. In addition, the constituent α and β phases within the prior-β grains change from lamellar colony to basket-weave, although no α′ martensite was detected in the microstructure. Such a phase inhomogeneity has also been reported in geometrically complex Ti−6Al−4V component produced by EB-PBF[18].

Supplementary Fig. 19 compares the tensile properties of Ti−6Al−4V produced by EB-PBF[17] and L-PBF to those of the newly developed alloys by L-PBF in this work. The L-PBF produced Ti−6Al−4V exhibits a dramatic variation of elongation to failure, but its yield strength remains almost unchanged. Ti−6Al−4V fabricated by EB-PBF shows significant variations of both yield strength and elongation to failure. Although in-situ martensite decomposition can be achieved in the EB-PBF produced Ti−6Al−4V, the heterogeneity in both prior-β grains and constituent phases cannot be eliminated and, as a result, the heterogeneity in

mechanical performance remains. In contrast, the newly developed alloys show less changes in both yield strength and ductility compared to Ti−6Al−4V fabricated by either L-PBF or EB-PBF, as can be expected from the homogeneous lamellar (α+β) microstructures (Supplementary Fig. 1d and Supplementary Fig. 11).

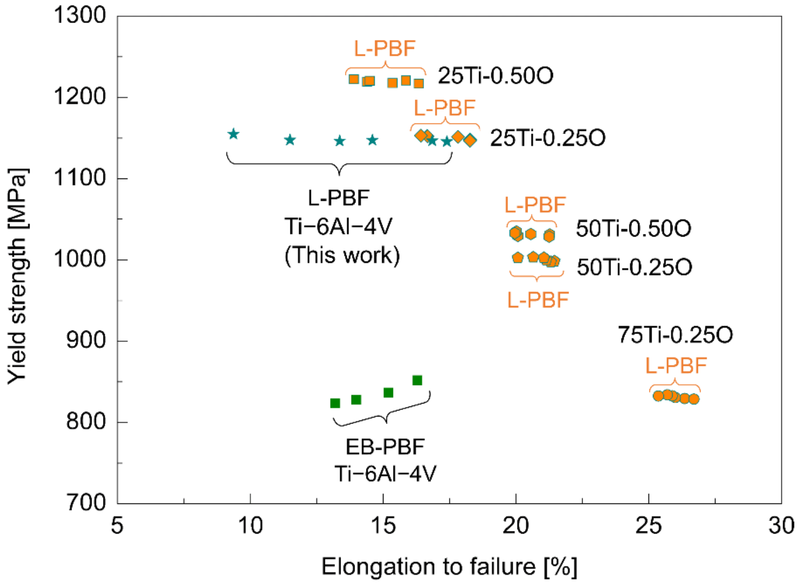

Supplementary Fig. 19 | Comparison of tensile properties of Ti−6Al−4V (by EB-PBF and L-PBF) and those of the newly developed alloys (by L-PBF). Note that the tensile properties of Ti−6Al−4V are collected from Fig. 1b in this work.

Supplementary Note 5 − The synergistic effect of CP−Ti and Fe_2O_3 additions on the phase homogeneity

To reveal the synergistic effect of CP−Ti and Fe_2O_3 on the phase homogeneity, we fabricated the parts using the feedstock with the sole addition of either CP−Ti or Fe_2O_3 to Ti−6Al−4V. Supplementary Fig. 20 shows the microstructures of (Ti−6Al−4V + 50 wt % CP−Ti) along the building direction. It is found that the top surface consists of relatively coarse α or α′ phases, without any detectable β phase. The phase width is notably larger than that of Ti−6Al−4V in the top surface (Supplementary Fig. 20a,b). In the lower region, α or α′ phases remain coarse but a small amount of β phases can be found at the interface of two α/α′ phases

(Position B). β phases become more evident as the build height lowers (Position C and Position D). Besides, the width of α or α′ phase varies along the building direction, spanning from 1.43 ± 0.66 μm in the top surface (Position A) to 0.54 ± 0.27 μm in the bottom region (Position D), as shown in Supplementary Fig. 20c. Therefore, the sole addition of CP−Ti to Ti−6Al−4V cannot eliminate the phase heterogeneity.

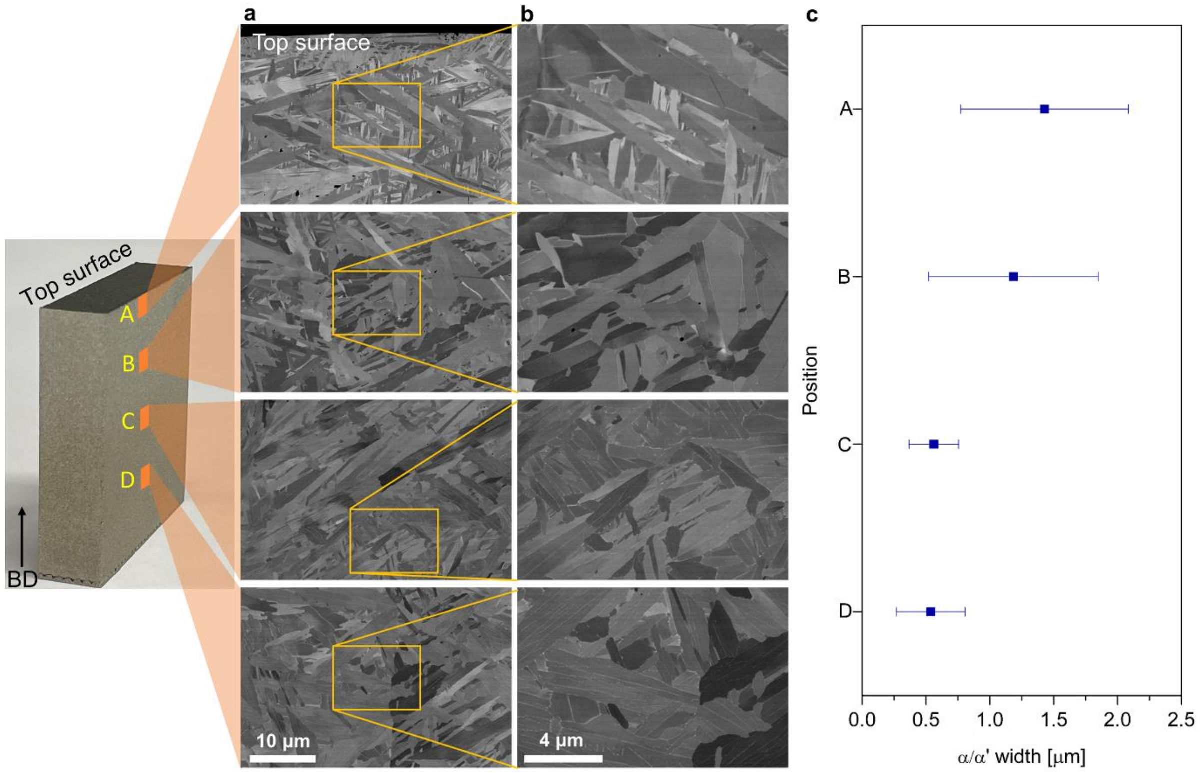

Supplementary Fig. 20 | Microstructures of (Ti−6Al−4V + 50 wt % CP−Ti) along the building direction. a, SEM-BSE images of microstructures in the 50Ti part at different locations along the building direction (BD). **b,** Higher magnification of the selected regions in **a**. **c,** the α/α′ width of at different positions of the part. The phase analysis was carried out using the ImageJ software.

Supplementary Fig. 21 shows the microstructural analysis of (Ti−6Al−4V + 0.25 wt % Fe_2O_3) along the building direction. Overall, the addition of 0.25 wt % Fe_2O_3 to Ti−6Al−4V results in a significant reduction in the phase width throughout the part compared with Ti−6Al−4V (Supplementary Fig. 1a,b). It is apparent that the internally twinned martensite

remains in the top region (Position A and Position B) and a fully lamellar (α+β) microstructure forms in the lower region (Position D), indicating that the sole addition of Fe_2O_3 to Ti−6Al−4V cannot eliminate the phase heterogeneity.

In summary, by comparing the microstructures of (Ti−6Al−4V + 50 wt % CP−Ti) (Supplementary Figs. 20a,b) and (Ti−6Al−4V + 0.25 wt % Fe_2O_3) to those of 50Ti−0.25O (Supplementary Fig. 11c,d), it suggests that CP−Ti and Fe_2O_3 have a synergy in their contributions to the homogeneous lamellar (α+β) microstructures.

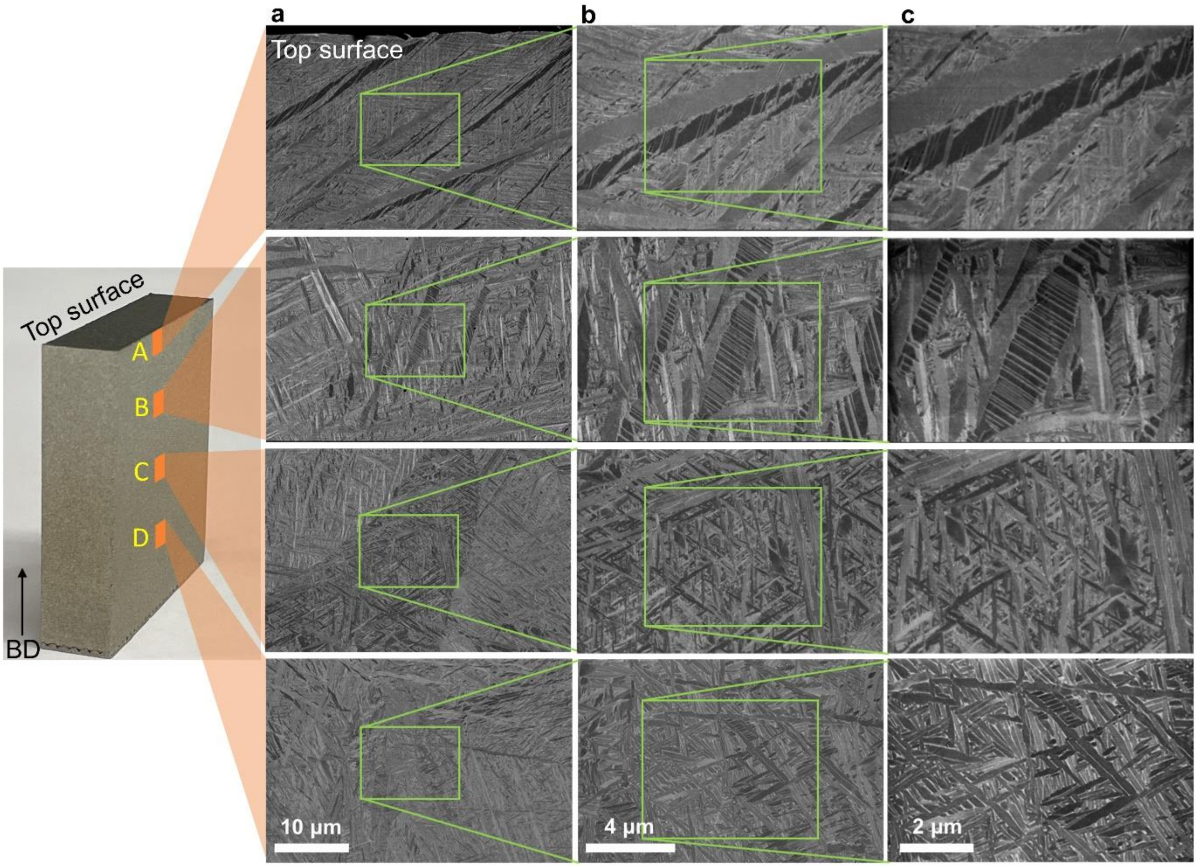

Supplementary Fig. 21 | Microstructures of (Ti−6Al−4V + 0.25 wt % Fe_2O_3) along the building direction. a, SEM-BSE images showing the microstructures in the fabricated part at different locations along the building direction (BD). **b,** Higher magnification of the selected regions in **a**. **c,** Higher magnification of the selected regions in **b**.

Supplementary Note 6 – The phase transformation pathways for L-PBF produced Ti−6Al−4V and the newly developed alloys

Based on the detailed microstructural analysis, a schematic diagram is presented to compare the phase transformation pathways for Ti−6Al−4V and the newly developed alloys during L-PBF. As shown in Supplementary Fig. 22, the L-PBF process involves multi-track and multi-layer melting of the powder feedstock (Supplementary Fig. 22a). For a location (marked with J) in a single layer of the fabricated part, it underwent multiple thermal cycles (marked with thermal cycling I), due to the track-by-track melting this layer. Subsequently, the layer-by-layer fabrication produces repeated thermal cycling (II, III and IV). The peak temperatures of such repeated thermal cycling decreases[19]. In the case of Ti−6Al−4V (Supplementary Fig. 22c), owing to the high cooling rate at the martensite start temperature (M_s), acicular α′ martensite transforms from the parent β phase (A→B in Supplementary Fig. 22c). There might be a small amount of retained β phases due to rapid solidification. It has been shown that such a small amount retained β phase cannot be detected by conventional techniques such as XRD and TEM but can be only evidenced by neutron diffraction[20]. As the successive layers are fused, the associated thermal cycling acts as an intrinsic heat treatment, thereby resulting in a partial decomposition of α′ (B→C in Supplementary Fig. 22c). It is found that α′ martensite decomposition in Ti−6Al−4V can take place at temperature as low as 400 ^{o}C[21]. When the accumulated thermal cycling effect is sufficiently strong and long, full lamellar (α+β) microstructures can be achieved (C→D in Supplementary Fig. 22c). In contrast, the fast diffusion of Fe in the newly developed alloys speeds up the formation of full lamellar (α+β) microstructures (A→D in Supplementary Fig. 22c), without the need of substantial thermal cycling. Therefore, homogeneous lamellar (α+β) microstructures are obtained throughout the fabricated part.

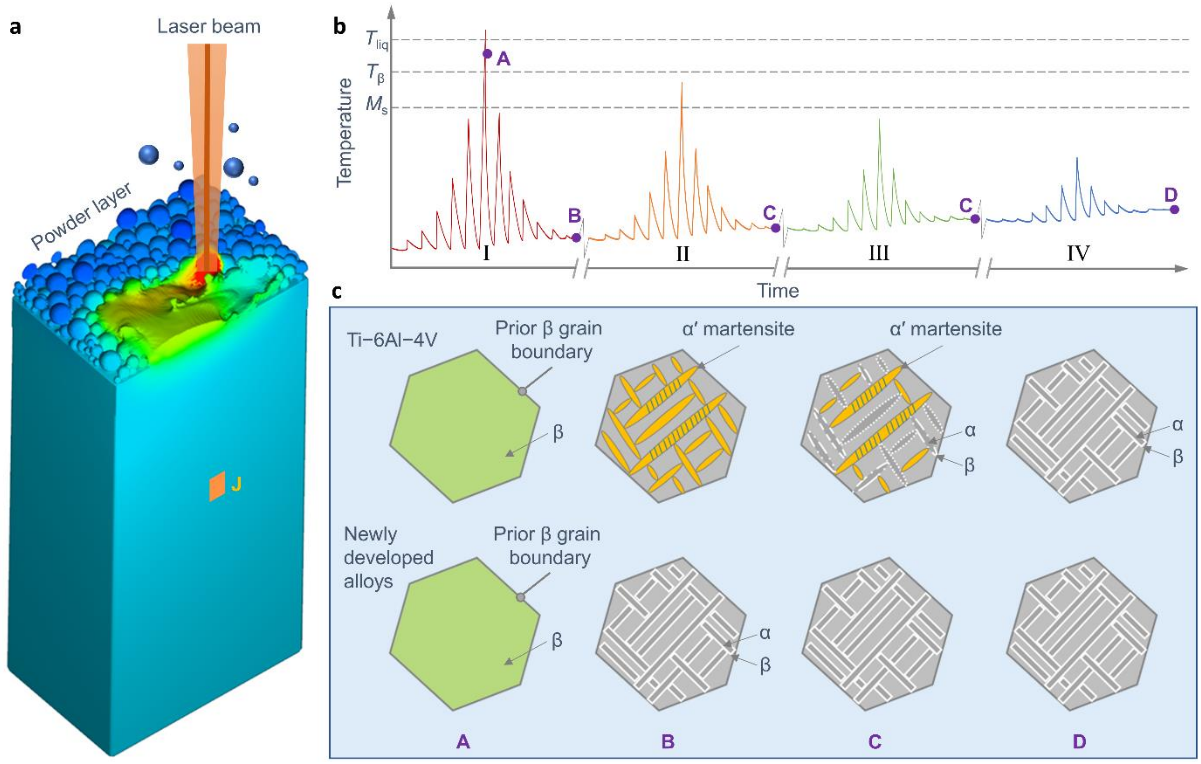

Supplementary Fig. 22 | Schematic illustration of phase transformation pathways for L-PBF produced Ti−6Al−4V and the newly developed alloys under the thermal cycling. a, The L-PBF process. **b**, The thermal cycling of a location (marked with orange box J) experienced during L-PBF due to the track-by-track and layer-by-layer fabrication. **c**, The sequence of phase transformation at different stages (A, B, C and D marked in **b**) in Ti−6Al−4V and the newly developed alloys.

Supplementary References

1. Liu, S. Y. & Shin, Y. C. Additive manufacturing of Ti6Al4V alloy: A review. *Mater. Des.* **164**, 1–28 (2019).
2. Kok, Y. et al. Anisotropy and heterogeneity of microstructure and mechanical properties in metal additive manufacturing: a critical review. *Mater. Des.* **139**, 565–586 (2018).
3. Voisin, T. et al. Defects-dictated tensile properties of selective laser melted Ti−6Al−4V. *Mater. Des.* **158**, 113–126 (2018).

4. Noell, P. J., Carroll, J. D. & Boyce, B. L. The mechanisms of ductile rupture. *Acta Mater.* **161**, 83–98 (2018).

5. Moridi, A. et al. Deformation and failure mechanisms of Ti-6Al-4V as built by selective laser melting. *Mater. Sci. Eng. A* **768**, 138456 (2019).

6. Krakhmalev, P. et al. Deformation behavior and microstructure of Ti6Al4V manufactured by SLM. *Phycs. Proc.* **83**, 778–788 (2016).

7. Simonelli, M. et al. The influence of iron in minimizing the microstructural anisotropy of Ti-6Al-4V Produced by Laser Powder-Bed Fusion. *Metall. Mater. Trans. A* **51**, 2444–2459 (2020).

8. Narayana, P. L. et al. Microstructural response of β-stabilized Ti−6Al−4V manufactured by direct energy deposition. *J. Alloys Compd.* **811**, 152021 (2019).

9. Easton, M.A., Qian, M., Prasad, A. & StJohn, D.H. Recent advances in grain refinement of light metals and alloys, *Curr. Opin. Solid State Mater. Sci.* **20**, 13−24 (2016).

10. Zhang, D. et al. Additive manufacturing of ultrafine-grained high-strength titanium alloys. *Nature* **576**, 91–95 (2019).

11. Maxwell, I. & I, Hellawell, A. A simple model for grain refinement during solidification, *Acta Metall.* **23**, 229–237 (1975).

12. Alabort, E. et al. Alloys-by-design: application to titanium alloys for optimal superplasticity. *Acta Mater.* **178**, 275–287 (2019).

13. Zhao, P. et al. Corrosion behavior of dual-phase Ti–6Al–4V alloys: A discussion on the impact of Fe content. *J. Alloys Compd.* **858**, 157708 (2021).

14. Dai, N. et al. Distinction in corrosion resistance of selective laser melted Ti-6Al-4V alloy on different planes. *Corros. Sci.* **111**, 703–710 (2016).

15. Yan, M. et al. Review of effect of oxygen on room temperature ductility of titanium and titanium alloys. *Powder Metall.* **57**, 251–257 (2014).

16. Saville, A. I. et al. Texture evolution as a function of scan strategy and build height in electron beam melted Ti-6Al-4V. *Addit. Manuf.* **46**, 102118 (2021).
17. Tan, X. et al. Graded microstructure and mechanical properties of additive manufactured Ti−6Al−4V via electron beam melting. *Acta Mater.* **97**, 1–16 (2015).
18. Wang, P. et al. Spatial and geometrical-based characterization of microstructure and microhardness for an electron beam melted Ti-6Al-4V component. *Mater. Des.* **95**, 287–295 (2016).
19. Pantawane, M.V. et al. Coarsening of martensite with multiple generations of twins in laser additively manufactured Ti6Al4V. *Acta Mater.* **213**, 116954 (2021).
20. Mengucci, P. et al. Solid-state phase transformations in thermally treated Ti–6Al–4V alloy fabricated via laser powder bed fusion. *Materials* **12**, 2876 (2019).
21. Xu, W. et al. Additive manufacturing of strong and ductile Ti−6Al−4V by selective laser melting via in situ martensite decomposition. *Acta Mater.* **85**, 74–84 (2015).
