# Supplementary material for: Designing against phase and property heterogeneities in additively manufactured titanium alloys
Source: Nat Commun. 2022 Aug 9;13:4660. doi: 10.1038/s41467-022-32446-2 (PMC9363443; doi:10.1038/s41467-022-32446-2)
Supplement: Supplementary file 3 — Description of Additional Supplementary Files [file 41467_2022_32446_MOESM3_ESM.pdf]

Description of Additional Supplementary Files

File Name: Supplementary Movie 1

Description: **Atom probe tomography reconstruction.**

This video shows the 3D views of the β phases in the samples with different Fe_2O_3 addition levels (**a,** 50Ti−0.25O, **b,** 50Ti−0.50O). The β phases are highlighted by isosurfaces at 1.5 at % Fe (**a**) and 4.0 at % Fe (**b**), respectively. Scale bar unit, nm.
